# Cellular and Molecular Immunology Approaches for the Development of Immunotherapies against the New Coronavirus (SARS-CoV-2): Challenges to Near-Future Breakthroughs

**DOI:** 10.1155/2020/8827670

**Published:** 2020-12-19

**Authors:** Juliana Gil Melgaço, Danielle Brito e Cunha, Tamiris Azamor, Andrea Marques Vieira da Silva, Luciana Neves Tubarão, Rafael Braga Gonçalves, Robson Q. Monteiro, Sotiris Missailidis, Patricia Cristina da Costa Neves, Ana Paula Dinis Ano Bom

**Affiliations:** ^1^Laboratório de Tecnologia Imunológica, Instituto de Tecnologia em Imunobiológicos, Bio-Manguinhos, Fundação Oswaldo Cruz (FIOCRUZ), Rio de Janeiro, Brazil; ^2^Laboratório de Bioquímica Estrutural, Departamento de Bioquímica, Universidade Federal do Estado do Rio de Janeiro (UNIRIO), Rio de Janeiro, Brazil; ^3^Laboratório de Trombose e Câncer, Instituto de Bioquímica Médica Leopoldo Meis, Universidade Federal do Rio de Janeiro (UFRJ), Rio de Janeiro, Brazil; ^4^Laboratório de Tecnologia de Anticorpos Monoclonais, Instituto de Tecnologia em Imunobiológicos, Bio-Manguinhos, Fundação Oswaldo Cruz (FIOCRUZ), Rio de Janeiro, Brazil

## Abstract

The severe acute respiratory syndrome caused by the new coronavirus (SARS-CoV-2), termed COVID-19, has been highlighted as the most important infectious disease of our time, without a vaccine and treatment available until this moment, with a big impact on health systems worldwide, and with high mortality rates associated with respiratory viral disease. The medical and scientific communities have also been confronted by an urgent need to better understand the mechanism of host-virus interaction aimed at developing therapies and vaccines. Since this viral disease can trigger a strong innate immune response, causing severe damage to the pulmonary tract, immunotherapies have also been explored as a means to verify the immunomodulatory effect and improve clinical outcomes, whilst the comprehensive COVID-19 immunology still remains under investigation. In this review, both cellular and molecular immunopathology as well as hemostatic disorders induced by SARS-CoV-2 are summarized. The immunotherapeutic approaches based on the most recent clinical and nonclinical studies, emphasizing their effects for the treatment of COVID-19, are also addressed. The information presented elucidates helpful insights aiming at filling the knowledge gaps around promising immunotherapies that attempt to control the dysfunction of host factors during the course of this infectious viral disease.

## 1. Introduction, Epidemiology, and Challenges to Near-Future Breakthroughs

In December 2019 in Wuhan, China, the first cases of a serious respiratory disease detected so far without etiology were reported, named by the World Health Organization (WHO) as “new corona virus 2019” and later defined as Coronavirus for Severe Acute Respiratory Syndrome 2 (SARS-CoV-2) causing coronavirus disease, termed as COVID-19 [1, 2].

The identification of the first patients reported with COVID-19 also identified a common history associated with the local market for seafood in Huanan, where wild animals are traded. The genomic characterization of the new virus identified a close similarity to two bat coronaviruses (SARS-CoV and pangolin coronavirus) linking the origin of SARS-CoV-2 with the wholesale market and indicating its zoonotic origin [1, 3].

SARS-CoV-2, responsible for the current pandemic declared on March, 11th by the WHO, has been overwhelming on a global scale, presenting high transmission rates with a large number of deaths, bringing severe effects to health services and economies worldwide. Until July, 20th, WHO reported 14,348,858 confirmed cases of COVID-19 and 603,691 deaths worldwide, reaching all six continents [4].

The SARS-CoV-2 virus belongs to lineage B of the genera *β*-coronavirus, belonging to the *Coronaviridae* subfamily. Other viruses of this *Coronaviridae* family such as HCoV-OC43, HCoV-229E, HCoV-HKU1, and HCoV-NL63, responsible for causing around 15% of common colds [5], are already known, in addition to the Severe Respiratory Syndrome Virus (SARS) and Middle East Respiratory Syndrome-related Coronavirus (MERS) responsible for causing the epidemics in 2002-2004 and 2012, respectively. Of these, SARS, now called SARS-CoV-1, belongs to the same lineage B and, therefore, presents greater epidemiological similarity to SARS-CoV-2. For this reason, it is being widely used in current studies, in an attempt to gain a better knowledge and understanding of this disease and also in the search for treatment and vaccine options for COVID-19 [6–8].

COVID-19 is a disease that may remain asymptomatic, or may present light symptoms, evolving to a more severe disease and can eventually lead to death. The role that asymptomatic individuals play in the spread of SARS-CoV-2 is not well described. Many studies present asymptomatic cases as probable sources of infection, playing an important role in the spread of these viruses [9]. Research estimates that these cases can represent 60% of all infections [10]. However, further studies should be carried out to assess both the amount and infectivity of viral load in asymptomatic individuals to better understand the course of the disease and the necessary methods to fight it [11].

The great challenge of this disease is the lack of knowledge about the SARS-CoV-2 virus, its adaptation, and its effects on the human body, especially as there is no effective drug, vaccine, or treatment available against COVID-19 until this date (July 2020). Therefore, intervention measures to contain the pandemic are limited to social behavioral interventions, such as the use of masks, social distancing, self-isolation, quarantine, and even blocking entire territories and communities, to contain or, at least, mitigate the burden of the ongoing pandemic [12].

The search for a vaccine or an effective treatment is evading laboratories around the world, and several articles have been published daily to share the knowledge obtained. This public health issue is affecting several sectors, mainly the social and economic sectors, causing governmental management to opt for a shorter period of isolation measures, and proceeding to a gradual reopening of the economy, returning to a “new normal.” This restart of activity is believed to cause more outbreak waves, due to increased contact rates [7].

So far, during the onset of COVID-19 symptoms, it has been known that a dysfunction of the immune response is part of the virus-host interaction process and can lead to disease severity and poor outcomes [13–16]. Protective immunity after the infection is not guaranteed. Despite the cross-reactivity with other human common cold coronaviruses in those with previous contact, protection against SARS-CoV-2 infection cannot be confirmed [17]. Furthermore, there is little information regarding immunity [1, 6], raising questions that still have no answers, such as which pathway in the immune response can be the key to attempt to control the immune dysfunction? How can immunotherapy be effective in all subjects affected?

After seven months from the first confirmed case of COVID-19, the knowledge about immune events during SARS-CoV-2 infection remains incomplete, and solutions to the main questions are urgently needed. In this review, we aim to gather information on the main immune responses described in the literature, as well as to offer insights into the promising immunotherapies that can be helpful for the scientific community thus assisting in the delivery of solutions for the urgently needed near-future breakthroughs.

## 2. Cellular Immune Response during COVID-19: What Is Known So Far

### 2.1. Humoral Response by B Cells

There is a great discussion regarding the adaptive immune responses to SARS-CoV-2, whether these are protective or pathogenic, or whether both scenarios are possible. The management and knowledge of immune response composition, kinetics, and magnitude can determine the real protection against this pathogen [17]. In this context, the search for correlates of protection biomarkers and reliable immunological parameters has become the key to eliminating this disease [18].

Knowledge of the nature and extent of B cell memory response becomes of great importance, given that the protection from reinfection has directed medical and social initiatives, including the search for vaccine strategies and the return to regular activities. Studies from countries that were in the epicenter of the pandemic showed that, despite a large number of identified cases, the percentage of people who developed antibodies to SARS-CoV-2 is less than expected, like 19.9% in New York City, 17.5% in London, and 11.3% in Madrid [19].

The B cell responses in COVID-19 occur concomitantly with T cell responses, around 1 week after the onset of symptoms. In general, the humoral response to SARS-CoV-2 is more evident between 10 and 25 days [18]. Patients infected with SARS-CoV-2 present the highest levels of IgM antibodies between days 11 and 25 and IgG antibodies between 14 and 25 days after infection [20–22].

CD4+ helper T cells are also known to play an important role in inducing antibody production by B cells during viral infections. During infection, follicular helper T cells (Tfh) are important and crucial for initiating the antibody response at the germinal center for affinity maturation and humoral memory response [23, 24]. In COVID-19, a drop on T cell numbers have been described in the acute phase and even in mild cases, without antibody production or diminishing of IgG antibody detection in the recovery phase [25, 26]. The reduction of virus-specific antibody production could be explained by any failure on the cell differentiation caused by SARS-CoV-2, mainly for CD4+ T cells on the germinative center, where autopsy studies showed lymphocyte depletion in the spleen and lymph nodes [17, 27, 28].

The remaining question is whether the generated antibodies confer long-lasting immunity. In fact, it seems that a subset of patients may not develop long-lasting antibodies to SARS-CoV-2 [29]. Due to the pandemic being a recent event, it is not possible to obtain this response yet; however, it can be predicted based on what is learned from the study of other human coronaviruses [30]. In general, studies have shown that the antibody response among coronaviruses is rarely detected in the acute phase of the disease (until the 7th day). The median time of detection was similar across different antibodies for SARS-CoV-1 (12 days) and SARS-CoV-2 (11 days), but longer for MERS-CoV (16 days) [8].

The comparisons are greater with SARS-CoV-1, which belongs to the same strain of beta-coronavirus [30]. Since 2002, studies have been carried out in order to better understand the activity of this virus. A three-year follow-up study shows that IgM seroconversion was related to a peak on the 15th day, and the performance of IgG and neutralizing antibodies against SARS-CoV-1 peaked at month 4 for both, decreasing over time and becoming almost undetectable in up to 25% of individuals after 36 months [31]. Analysis of IgG memory cells specific for SARS-CoV-1 protein S indicates a progressive cell decrease of ~90% from 2 to 8 months after infection [32]. A follow-up study of 34 healthcare professionals infected with SARS-CoV-1 also shows that virus-specific IgG decreased after several years; however, after 12 years of infection, the authors observed specific detectable IgG [33]. These and other studies of human coronaviruses demonstrate a tendency of antibody response against these viruses to decrease over time. Some studies and mathematical models point to a short duration of immunity after infection by SARS-CoV-2. It was observed that IgG and neutralizing antibody levels in convalescent patients decrease within 2 to 3 months after infection. In another study carried out with 8 convalescent patients, 50% showed a decrease of neutralizing antibody levels in approximately 6 to 7 weeks after disease onset [26]. Further studies will be needed overtime to determine the degree of protection to SARS-CoV-2.

Although many discussions about cross-immunity are taking place, they are still at odds. Previous studies point to a cross-reaction between viruses of the coronavirus family, but reactivity between endemic HCoVs, SARS-CoV-1, and MERS viruses was minimal. Evaluation of plasma from patients with COVID-19 showed a cross-reaction with the S and N SARS-CoV-1 proteins and the S protein of MERS-CoV, but no cross-reaction was observed for the receptor-binding domain (RBD) of these viruses. In addition, it was observed that the plasma of patients with COVID-19 did not neutralize SARS-CoV-1 or MERS-CoV, suggesting that antibody-mediated neutralization is virus-specific and is probably driven by the binding of epitopes within the RBD [34]. Another study of 149 convalescent COVID-19 patients by Robbiani et al., demonstrated that despite the low activity of neutralizing antibodies in most plasmas of these individuals, recurrent anti-RBD SARS-CoV-2 antibodies with potential neutralizing activity were detected, suggesting RBD as the efficiency target for neutralizing the viral disease [35]. However, other studies showed that RBD structures of SARS-CoV-1 and SARS-CoV-2 present some differences, and therefore, only a few antibodies against SARS-CoV-1 were able to neutralize SARS-CoV-2 [36].

The correlation between the level of antibodies produced and the severity of the disease even for other coronaviruses is still unclear [8]. Preliminary studies with SARS-CoV-2 indicate that the viral load and disease severity may be related to the magnitude of IgG antibodies [33, 37]. While IgM appears both in severe and nonsevere cases at the same time, IgG appears 2 to 3 days earlier in severe cases [37, 38]. Moreover, S protein mutations may be responsible for viral resistance to antibody recognition and consequent aggressiveness [29]. Caution is needed since the total measurable antibody is not precisely the same as protective. SARS-CoV-2 RBD of the S protein (RBD-S) is highly immunogenic. Antibodies that bind to this domain, blocking the interaction with Angiotensin-Converting Enzyme 2 (ACE2) (the host's input receptor) are characterized as potentially neutralizing. Virus-neutralizing antibodies are presumed to be an important mechanism of action in COVID-19 since high titers of these antibodies have been related to severe cases of the disease. In addition, some patients present low or very low titers of COVID-19 neutralizing antibodies up to 2 weeks after hospital discharge [38, 39].

Studies report successful plasma convalescent therapy as a source of therapeutic polyclonal antibodies, indicating monoclonal antibodies as an excellent therapeutic possibility [40]. However, a few researchers are concerned about the possibility of antibody-dependent enhancement (ADE), where the preexistence of anti-SARS-CoV-2 antibodies could exacerbate the immune response to SARS-CoV-2. One of the findings that support this hypothesis is that patients who developed responses against protein S earlier are related to the worse prognosis of the disease [41]. Another possibility is that the virus-specific nonneutralizing IgG could facilitate the entry of viral particles into cells that express Fc receptors (FcR), leading to the inflammatory activation of these cells, such as macrophages and monocytes [30]. This event was also observed during the SARS-CoV-1 epidemic, where deceased patients presented much higher neutralizing antibody titers when compared to patients who recovered [41]. So far, there is no evidence that antibodies developed for SARS-CoV-2 present such characteristics.

Studies with animal models showed that serum from rats vaccinated with SARS-CoV-2 RBD [42], as well as monkeys immunized with an inactivated SARS-CoV-2 vaccine candidate [43], were successful in obtaining antibodies and showed no ADE evidence. However, this hypothesis must be validated by further scientific research and also by longer time intervals in order to assess an effective and safer concentration of antibodies. With regard to vaccine aspects, the role of follicular helper T cells in the production of antibodies should also be considered to guarantee comprehensive protection. Juno et al. in 2020 found that antigen-specific antibodies, memory B cells, and follicular helper T cells are elicited after SARS-CoV-2 infection and correlated to potent neutralizing responses, suggesting that T cells together with B cells should be investigated to evaluate the potency of antigen-based vaccines [44].

The immunological scenario regarding COVID-19 indicates a secondary role of humoral response in terms of effectiveness, suggesting the importance of innate and cellular response.

### 2.2. Cellular Immune Response Dysregulation: Innate and Adaptive Immunity

Some studies described that the acute phase of COVID-19 is divided into two aspects: benign and severe, and it is correlating with immune dysfunction alterations [13–16]. The literature showed that macrophages and neutrophils may be responsible for the high activation of inflammatory pathways, which exacerbates the production of proinflammatory cytokines and chemokines, leading to the “cytokine storm” phenomenon [13, 45, 46].

**Figure 1:**
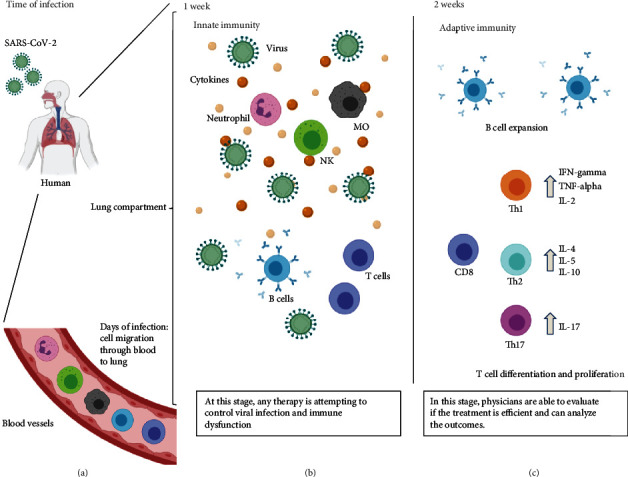
Immune response during COVID-19. (a) After SARS-CoV-2 contact with the respiratory tract of a human, susceptible cells are infected by entry through ACE2 or CD147 surface receptors. Once in the lung compartment, the first line of defense made of dendritic cells and other cells, such as macrophages, are able to recruit other immune cells. Then, cell migration through blood vessels are directed to the lung compartment, where cell activation occurs, initiating the inflammation process on the first week of signals and symptoms (b), inducing natural killer cell (NK), neutrophil, and macrophage (MO) activation in the lungs, causing the phenomenon called “cytokine storm.” In the mean time, B cells are producing antibodies, and immature T cells are also activated attempting to control the viral infection, although apoptotic events can also occur, reducing the number of T cells in the lungs and in the peripheral blood. After that, in two or more weeks (c), adaptive immune response is raised to specific IgG and neutralizing antibodies to contribute to the recovery and viral elimination. At this time, CD8 T cells are activated as cytotoxic phenotype, with the same aim, driving towards virus elimination. Meanwhile, the differentiation of CD4 T cells can trigger for more help for viral elimination by Th1 cells, but it can also lead to more lung damage by inflammatory phenotypes, such as Th2 and Th17 cells. Physicians and clinicians should evaluate each case to introduce the immunotherapies attempting to control the immune dysfunction. Created with BioRender.com.

A highly activated innate immune response may be the cause of disease progression, as well as poor outcome, including death [2, 47, 48]. Wang et al. in 2020 showed that natural killer (NK) cells were decreased in peripheral blood from COVID-19 patients, which may be related to these cells' function in the lung compartment, contributing to greater inflammatory responses [6, 45]. T lymphocytes are also involved in the immune imbalance during COVID-19, with a massive decrease of CD4 and CD8 total numbers in the initial phase [37], and with CD4 T helper cells found in the pulmonary tract [45], showing a possible effect of SARS-CoV-2 on lymphocyte lysis and an important function of CD4 T helper cells in the lung damage in the acute phase [2, 45] (Figure 1).

Wang et al. in 2020, showed that SARS-CoV-2 infects T cells through the spike S protein, and the entry is facilitated not just by the ACE2 receptor, but also by the CD147 expression at the cell surface [49, 50], which may contribute to immune cells carrying the virus to other organs.

**Figure 2:**
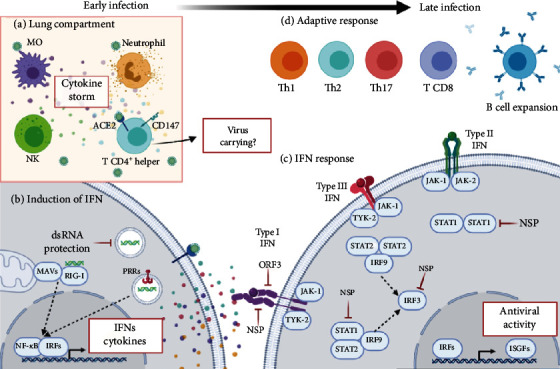
Summary of immunopathogenesis in early and late SARS-CoV-2 infections. (a) Early in the lung compartment, activated macrophages, neutrophils, NK cells, and T CD4+ helper cells release an exacerbated production of an immune mediator called “cytokine storm” that contributes to lung damage. Entry of T CD4+ helper cells is facilitated not just for ACE2 and CD147 receptors, which may contribute to carrying virus to other organs. (b) Still in the early phase, virus-cell interaction leads to an innate cellular response. During viral replication, dsRNA is recognized by RIG-I that interacts with MAVs leading to the activation of NF-*κ*B transcription factors and IRF which induce the expression of IFNs and proinflammatory cytokines. However, SARS-CoV-2 can evade PRRP recognition shielding dsRNA by membrane-bound compartments that form during viral replication. (c) Following IFN release, the first activated pathway is type I IFN. IFN-*α*/*β* interaction with IFNAR receptor activates TYK-2 and JAK-1. This interaction could be blocked by SARS-CoV-2 by viral ORF3 and NSP. Virus can interact directly with STAT1 inhibiting interaction with STAT2 and IRF9. Next, type III IFN (IFN-*λ*) interaction with IFNLR/IL-10R leads to activation of TYK-2 and JAK-1, followed by STAT2-IRF9 interaction. Both type I and III IFN pathways activate IRF3, which is a target of viral NSP proteins. The last IFN pathway activated after SARS-CoV-2 infections is type II IFN (IFN-*γ*), related to adaptive responses. IFN-*γ* interaction with IFNGR receptor activates JAK-1 and JAK-2, followed by STAT1, target of viral evasion by NSP. All IFN pathways lead to expression of antiviral ISGFs. (d) Finally, the cellular profiles change to adaptive immune response with B cell expansion and specific antibody production. At this time, CD8 T cells are activated as a cytotoxic phenotype, and CD4 T cells differentiate in Th1 cells that help viral elimination, as well as inflammatory phenotypes Th2 and Th17. Created with BioRender.com.

**Figure 3:**
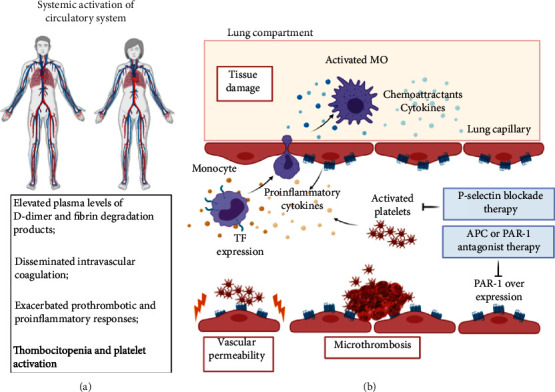
Hemostatic disorder in SARS-CoV-2 infection. (a) In COVID-19 cases, there occurs a systemic activation of the circulatory system as a reflection of what occurs in the lungs. (b) In the lung compartment, monocytes expressing TF perform an intense diapedesis under stimuli of proinflammatory and chemoattractant cytokines, infiltrating activated macrophages in the lung that lead to tissue damage. The high production of proinflammatory cytokines, as well as vascular permeability and microthrombosis events are prompt to overexpress PAR-1 receptors and abnormal platelet activation. Overexpression of PAR-1 can be blocked with APC or PAR-1 antagonist therapy, while abnormal platelet activation can be avoided by P-selectin blocker therapy. Created with BioRender.com.

Since the beginning of the pandemic, the event termed as “cytokine storm” has been the focus of several studies and cytokine and chemokine production has been used as a predictor of severity or death, or as means to evaluate therapeutic targets [13, 46, 51]. Cytokines/chemokines IL-1*β*, IL-6, IL-7, IL-8, IL-9, IL-10, CXCL10, CCL2, CCL3, CCL4, TNF-*α*, IL-12, and IL-17, have been described in high levels on plasma/serum samples from patients with severe COVID-19 and even higher in patients prior to death [40]. Natural killer cells, macrophages, and immature T cells can produce these components, being chemoattractant to neutrophils in the lung compartments, inducing a reduction of blood vessels, and subsequently, in the blood circulation, leading to oxygen impairment and facilitating thrombosis activation [45, 47, 52] (Figures 1–3).

The innate immune response is the organism's first line of defense, and an effective response depends on the balance between activation and regulation of the patient's immune system. Among these, type I and III interferons (IFNs) are considered the most important for antiviral defense, which mediate the onset of the innate immune response. Upon activation of pattern recognition receptors (PRRs), downstream signaling cascades trigger the secretion of cytokines [53]. Early on, coronavirus (CoV) prevents recognition by PRRs, and dsRNA is first shielded by membrane-bound compartments formed during viral replication of SARS-CoV-1 [54].

Interferon cytokines affect several other processes, including those regulating cell growth, differentiation, and apoptosis, as well as the modulation of the immune response. The main receptor is retinoic acid-inducible gene I (RIG-I) that detects viral RNA and interacts with mitochondrial antiviral signaling proteins (MAVs), leading to the activation of nuclear factor kappa B (NF-*κ*B) transcription factors and interferon regulatory factor family (IRF) which induces the expression of IFNs and proinflammatory cytokines. Studies using BALB/c mice showed that early IFN-I response reduced SARS-CoV titer and regulated inflammation and mild clinical disease [55]. Furthermore, SARS-CoV-2 is sensitive to IFN-I pretreatment [56] as demonstrated by *in vitro* assays. After binding to their receptors, IFN-I and III initiate the activation of Janus kinase 1 (JAK-1) and tyrosine kinase 2 (TYK-2). Phosphorylation recruits the signal translation and transcription activators (STAT1 and 2), forming a heterodimer of interferon regulatory factor (IRF). This complex forms the gene factor stimulated by interferon (ISGFs) that translocate to the nucleus, binding to the IFN response element (ISRE), inducing the production of hundreds of ISGs [53].

Type I and III IFNs are activated after a viral infection, and several reports suggest redundant functions. However, the transcription of these genes is regulated over time, since type I IFNs are expressed and resolved quickly, while type III IFNs present late, sustained, and tissue-specific induction. On the other hand, type II interferon (IFN-*γ*) is synthesized almost exclusively by the activated natural killer (NK) cells and activated T cells, in response to virus-infected cells. It also plays a role in mediating protection against viral infections, especially long-term control of viral infection. Studies showed that high levels of IFN-*γ* were associated with greater viral load and lung damage [57]. In contrast, suppressed IFN-*γ* production by CD4+ T cells might be correlated with the disease severity of COVID-19 [17] (Figure 2). However, SARS-CoV-2 present several evasion mechanisms of the innate immune response, and the genome viral load encodes a protein open reading frame (ORF3b) that inhibits the induction of type I interferon [58]. Analysis of the virus sequences isolated from two patients with severe COVID-19 disease showed a variant that further increased the ability of ORF3b to suppress interferon induction [59].

The other CoV, ORFs and nonstructural proteins (NSPs) interact directly or indirectly with PRR signaling cascades and IFN signaling pathway proteins, such as TBK1, IRF3, IFNAR1, and STAT1 [60]. Besides, CoV antagonizes RNaseL and STING, consequently impairing the development of an effective innate immune response [30]. The key point in SARS-CoV-2 infection could be the effect of viral evasion of the host defenses, related to the innate immune response. Thus, suppressing the production of INF cytokines at the right time could elicit an effective response against SARS-CoV-2 (Figure 2).

After 15-20 days of signals and symptoms, people who recovered from COVID-19 are presenting a reestablishment of their lymphocyte count, with an increase of CD4 T and CD8 T memory cells specific for SARS-CoV-2 protein [61, 62]. Nevertheless, an imbalance of total lymphocytes (CD3+) is not frequent for all patients, and this observation could be related to aging [15, 28]. Even with the current limitation of serological assays related to antibody detection, some studies have shown that memory T cells are emerging from peripheral blood to contribute to virus elimination and complete recovery [61–63] (Figure 1).

Despite those findings, there are challenges concerning the full protection from possible reinfection. The protection after infection and the presence of antibodies that do not guarantee the “immunity passport” is still poorly understood, as discussed [13, 62, 63]. Considering the above, more studies are necessary, and at the same time, new information is appearing daily with regard to immunity against the new coronavirus.

### 2.3. Hemostatic Disorders in COVID-19: A Consequence of Immune Events?

Patients with severe COVID-19 commonly exhibit signs of systemic activation of the coagulation system that relies on increased rates of thrombotic events and elevated plasma levels of D-dimer, a known marker of thrombogenesis [64–67]. In clinical observations, nonsurvivors present significantly higher levels of D-dimer and fibrin degradation products as well as longer prothrombin and activated partial thromboplastin compared to survivors on admission, thus meeting the criteria for disseminated intravascular coagulation [57, 67]. The mechanisms of COVID-19-driven coagulopathy are not fully understood but are possibly associated with the excessive immune/proinflammatory response [13, 68], which ultimately leads to the activation of several prothrombotic pathways [69, 70]. Therefore, there is strong evidence that the exacerbated procoagulant responses account for organ failure and the increased mortality in critically ill COVID-19 patients [71]. In this context, treatment with anticoagulants may improve the progress of severe COVID-19 [72, 73].

The crosstalk between inflammation and coagulation has long been described [74]. One of the major sources of these cytokines is activated monocytes/macrophages and neutrophils (Figure 3). During the infection, high plasma levels of CCL2 and CCL3, potent chemoattractants for monocytes, are noted [75]. Moreover, CCL2 and CCL7 are reported in bronchoalveolar lavage fluid from severe patients [76]. Therefore, these cells infiltrate massively into the lungs, as demonstrated in fatal cases of COVID-19 [36, 47].

Adding to that, dysregulated T cells secrete GM-CSF, which, together with IL-6, turn infiltrated monocytes into activated CD14+CD16+ macrophages [77]. These cells, still inside capillaries, probably express Tissue Factor (TF), as demonstrated largely in a number of viral infections, such as HIV and dengue, as well as microbial and other types of sepsis [78–80].

In the absence of tissue damage, TF expressed by activated monocytes initiates TF-dependent coagulation with the conversion of prothrombin to thrombin (Coagulation Extrinsic Pathway), in an uncontrolled manner, leading to disseminated intravascular coagulation. An increase in Protease-Activated Receptor 1 (PAR-1) expression in endothelial cells after viral infections is one of the consequences observed [81]. Although the main function of thrombin is to promote the formation of clots through the activation of platelets and the conversion of fibrinogen to fibrin, this protease exerts several cellular effects and may increase the inflammation process through the activation of PAR receptors, mainly PAR-1.

The activation of PAR-1 in endothelial cells promotes proinflammatory responses, resulting in the rupture of the endothelial barrier and an increase of cell permeability. [82]. These defects in procoagulant-anticoagulant mechanisms during inflammation may be responsible for predisposition of the development of microthrombosis, disseminated intravascular coagulation, and multiple organ failure in patients with severe COVID-19 pneumonia and in nonsurvivors [83] (Figure 3).

On the other hand, PAR-1 might be activated by the anticoagulant serine protease, activated protein C (APC), which binds to its coreceptor, endothelial protein C receptor (EPCR), and exerts cytoprotective and anticoagulant responses. Recombinant APC has been used as a drug to treat severe sepsis, a pathological condition characterized by exacerbated clotting and inflammation responses. However, the mechanism by which APC-activated PAR-1 promotes cytoprotective responses is still poorly understood [82]. In this context, PAR-1 antagonists and other coagulation protease inhibitors, in addition to modulators of the protein C pathway, may play an important role in the treatment of critically ill patients with COVID-19 [84].

Still, in the context of hemostatic disorders, platelets are also an important hub of classical crossover between the inflammatory and coagulation process [85–87]. Lungs are responsible for approximately 50% of platelet production and a site of mature and immature megakaryocytes [88], enhancing the implication of these anucleated cells in COVID-19 outcomes. From a clinical point of view, thrombocytopenia has been demonstrated in approximately 20% of in-hospital COVID-19 cases, with a strong association with thrombocytopenia and COVID-19 mortality. This event is most likely due to platelet consumption leading to the formation of pulmonary thrombi, which highlights the importance of monitoring platelet count during hospitalization [89, 90]. Besides, low count leads to platelet deposition in damaged pulmonary blood vessels in SARS patients [91, 92].

It has been described that activated platelets play a critical role in hemostasis and in coagulation, as well as in angiogenesis, inflammation, and even immune response, as demonstrated in other viral infections, like dengue and influenza. This activation occurs via P-selectin/prostaglandin-1 (PGL1), given viral engulfment and virus-platelet interaction [93]. In this way, a platelet RNA sequencing study of 25 COVID-19 patients demonstrated that platelets do not express the ACE2 receptor, although mRNA from the SARS-CoV-2 N1 gene was detected in platelets from 2/25 COVID-19 patients, suggesting that platelets may take-up SARS-CoV-2 mRNA independent of ACE2. Furthermore, resting platelets from COVID-19 patients increased P-selectin expression, both basally and upon activation [94].

With regard to immunological responses, platelets present multiple immune receptors, such as immunoglobulin or complement receptors and Toll-like receptors (TLRs) [85, 94], and the capacity of NRLP-3 inflammasome activation and production of cytokines IL-1*β* and TGF-*β* [95–97]. These functions are achieved through direct interaction with endothelial cells, as well as monocytes, lymphocytes (platelet-leukocyte aggregates (PLA)) and neutrophils (platelet-neutrophil aggregates (PNA)) in a process regulated by P-selectin [93, 98, 99]. PLA formation, specifically with monocytes, induces fibrin clot formation via interaction of PGL1 with tissue factors on the platelet surface [98], reduced IL-17 and IFN production when platelets adhere to CD4+ T-cells [99], and cross-presenting of antigens to CD8+ T cells given platelet expression of the major histocompatibility complex class I (MHC-I) [100]. The formation of PNA leads to C3 release from platelets and formation of Neutrophil Extracellular Traps (NETs), a mechanism that tightly regulates host immune responses and complement system responses, but can also promote thrombosis and damage lung capillaries [101, 102].

In COVID-19 patients, it has been demonstrated that circulating platelet-neutrophil, platelet-monocyte, and platelet-T-cell aggregates were all significantly elevated, enhancing the hypothesis that P-selectin blockade may be warranted in treating COVID-19 patients [94, 103, 104] (Figure 3).

## 3. Immunotherapeutic Insights: Important and Promising Breakthroughs

In fact, there are still no therapeutic agents for the effective treatment of COVID-19. Immunotherapy is described as a helpful tool to attempt to control immune dysfunction. However, to this moment, therapeutic intervention has been limited to severe cases, in order to minimize the risk of death, and also in combined therapies. Beyond therapies addressed to severe cases, it should be relevant to consider the differences on gender, age, pregnant condition, diabetes, hypertension, autoimmune diseases, heart diseases, cancer, and obesity during nonclinical and clinical studies, as well as the management for mild cases to avoid the worst outcome, since some comorbities are under risk of fatal COVID-19 [48, 105–107]. Actually, the surveillance of the immune events and their behavior should also be included in the guidelines for immunotherapies to treat COVID-19, once the immune response is crucial to the clinical evolution of the patient (Figure 1).

It is worth noting that some potential immunotherapies are very specific when targeting immune events and pathways, and they are designed to avoid adverse events, to be used exclusively to minimize the inflammation process induced by the viral disease, such as COVID-19. Therefore, immunotherapy approaches are developed to be useful in any situation, regardless of comorbity conditions. In this manuscript, we summarize some commercially available immunotherapeutic components with their mode of action and immunological impact in attempting to control the immune dysregulation during COVID-19, already used in clinical trials (human) (Tables 1(a) and 1(b)) and nonclinical studies (*in vitro* and *in vivo* animal models) for treatment purposes (Table 2).

Our search data was based on available findings up to July 20th, 2020. Furthermore, the number of registers with status “completed” or “terminated” (https://clinicaltrials.gov) was considered in order to determine clinical trials with results disclosed. Ongoing clinical trials were set up with a number of registers under “recruiting” status (https://clinicaltrials.gov). In addition, the clinical trials with preliminary results in humans in the preprint platforms and/or already published and/or recommended as treatment by published manuscripts were also considered. Nonclinical studies were classified as bioinformatic tools, cell cultivation assays, or animal models, with findings in the preprint platforms and/or already published with SARS-CoV-2, respiratory viral infections, or other diseases with similar immunopathological disorders, with recommendations to COVID-19 treatment by authors, or registration as a clinical trial with status varying from “recruiting,” “completed,” or “terminated.” The search for literature information was performed in digital medical science libraries, such as Google Scholar, SciELO, PubMed, bioRxiv, and medRxiv. From the bibliography found, only manuscripts with full text available were considered. The keywords used were severe acute respiratory syndrome, SARS-CoV-2, COVID-19, immunotherapy, immunomodulators, immunology, and therapy.

Considering the need for solutions and the speed of results, there are clinical trials approved, but without disclosure of preliminary results. According to these findings, we have been able to also show immunomodulatory products currently in test with the mode of action, purposes, and possible benefits to humans used in the treatment of COVID-19.

### 3.1. Clinical Trials: The State of the Art about the Tested and Ongoing Immunotherapies

#### 3.1.1. Antibody Plasma

Neutralizing antibodies from convalescent plasma have been used as an alternative to suppress viremia of SARS-CoV-2 in hospitalized patients, shortening the hospital stay, thus reducing mortality rates [108]. There is a risk that other infections will be installed through blood transfusion if the pathogen is not screened according to health practice guidelines and recommendations [108, 109]. Another fact regarding convalescent plasma is related to the possibility of applying it to a large number of patients, but it has been useful for the treatment of a few COVID-19 severe or critical patients [41, 110]. It is noteworthy that convalescent plasma could help the immune system to raise virus-specific T cells and improve viral elimination [30].

Passive immunity through intravenous gammaglobulin (IVIG) has been used widely for inflammation-related diseases, supplying the production of antibodies, suppressing inflammatory cytokines, and modulating T cell responses. This treatment has been used for COVID-19, not only using purified human plasma but also using purified plasma from animals. However, adverse events can occur, such as renal failure, and therefore, this treatment should be addressed with caution [41, 110] (Table 1(a)).

#### 3.1.2. Antiviral Compounds with Immunomodulatory Effect

Chloroquine (CQ) and hydroxychloroquine (HCQ) have been highlighted as important components to treat COVID-19. Both drugs have been used for autoimmune disorders, such as rheumatologic diseases with immunomodulatory effects, where they can enhance regulatory T cell activity and attenuate the “cytokine storm” [111]. *In vitro* studies with SARS-CoV-2 showed that these drugs inhibited endosomal TLR7 and TLR9 directly inducing endosomal maturation, thus driving the viral particles to lysosomes. However, CQ and HCQ already used on clinical trials show some controversial results with respect to the benefits for patients, demonstrating no efficiency on clinical results for COVID-19 treatment [30, 111] (Table 1(a)).

Ivermectin, an FDA-approved broad-spectrum antiparasitic drug has been reported for its antiviral activity against SARS-CoV-2 when used through cell cultivation [112]. This drug plays an important role in paralyzing parasites and in viral replication mechanisms, described already for several viruses [113]. Ivermectin was associated with lower mortality during the treatment of COVID-19 [114]. Additionally, an immunomodulatory effect of this drug in animal models and humans has been shown, including an increase of antibody production and leukocyte count [113, 115, 116]. Therefore, the immunomodulatory effect of ivermectin in COVID-19 should be better explored, though for now there are no changes in the evidence of immune response during COVID-19 described yet.

Oseltamivir is a very well-known drug to treat influenza virus infection, demonstrating effectiveness in reducing the viral load as well as inflammation, morbidity, and innate immune responses, and affecting the magnitude of the effector CD8+ T cell responses. Results on the immunomodulatory effects of Oseltamivir were observed in animal models and humans [117, 118]. Oseltamivir was also able to reduce the number of days of hospitalization for patients with COVID-19 [119] (Table 1(a)).

Atazanavir, an antiviral compound with antiretroviral protease inhibitor activity used in HIV patients, was able to reduce SARS-CoV-2 replication in tumor cell lines as well as in decreasing proinflammatory IL-6 and TNF-*α* levels in the supernatant of peripheral blood mononuclear cells in cell culture with the virus [120]. However, the immunomodulatory effect levels of IL-6 and TNF-*α* could be explained by the reduction of the viral load using Atazanavir *in vitro*. Now, there is an ongoing clinical trial testing this drug on COVID-19 patients (Table 1(b)).

#### 3.1.3. Cytokine-Directed Therapy

Interferons type I (alpha)/(beta) were widely used to treat several viral infections worldwide, due to their nonspecific antiviral ability, participating in the first line of defense [53]. Normally, interferon type I is used in combined therapies for viral infections [53, 121, 122]. Recently, Hadjadj et al. observed that interferon type I was impaired in the early acute phase of COVID-19 patients [122]. Robust IFN-I responses have also been observed in the respiratory tract and peripheral blood of patients with severe COVID-19 with high levels of interferon-stimulated genes (ISGs) along with high proinflammatory responses [123, 124]. Hung et al. in 2020 showed preliminary results from a phase 2 clinical trial with COVID-19 patients using 0.25 mg of commercial interferon beta-1b combined with other antiviral drugs (lopinavir/ritonavir/ribavirin). This combination was able to reduce the duration of viral shedding and days of hospitalization and mitigation of symptoms [125]. In a multicenter clinical trial, with 446 COVID-19 patients in Hubei, China, Wang et al. in 2020 demonstrated that early use of IFN-*α* decreased mortality, but its late use increased mortality and delayed recovery. In this way, the use of IFN therapy in COVID-19 needs precision medicine approaches, considering the favorable antiviral activity of IFN together with other direct antiviral therapies in early/mild infection and enhancement of harmful cytokine storm events in severe/late infections [126].

#### 3.1.4. Inhibition/Blockade of Immune Response Pathways

Some studies have been directed at better exploring the role of corticosteroids as a means to impair the immune cell response during SARS-CoV-2 infection [51]. However, some caution is needed since corticosteroids can induce the “shutdown” of the immune cell response and increase the viral infection process, raising the viral load and lysing lung cells [127, 128]. Corticosteroids can induce cell death and impair cytokine production by macrophages, neutrophils, and T cells. This component can promote the production of annexin 1, leading to the inhibition of the phospholipase A2 expression, responsible for pain and inflammatory events. Furthermore, corticosteroids can increase the expression of inhibitory proteins, leading to NF-*κ*B inactivation, reducing the transcription factors involved in Th2 type cytokine expression [129, 130].

Despite possible issues that could complicate the viral infection, a corticosteroid compound, Dexamethasone, has been described as a more successful therapy used until now for COVID-19 treatment in patients in intensive care units (ICU) with a severe grade of the disease, reducing the number of deaths [131–133]. Corticosteroids such as Methylprednisolone were also found to help in the treatment for COVID-19 [134] and other respiratory infections, including influenza [135], reducing the mortality rates for hospitalized patients [136].

As described here, hyperinflammation caused by SARS-CoV-2 infection leads to the “cytokine storm” [30, 111, 137]. In an attempt to control the exacerbation of lung inflammation, cytokine inhibitors and blockers of inflammatory pathways are in focus and under analysis to be used as therapy, becoming promising for the treatment of COVID-19. Some ongoing clinical trials are testing the efficacy of commercial anti-IL-6R (Tocilizumab/Actemra; Sarilumab/Kevzara) and anti-IL-6 antibody (Siltuximab/Sylvant) on COVID-19 patients used in conjunction with other therapeutic combinations (antiviral drugs and oxygen therapy). One group has already described preliminary data in a small population (*n* = 20), showing that patients recovered lymphocyte counts (52.6%), in which 95% of them presented a resolution of lung opacities on chest computed tomography as well as being discharged from the hospital, using Tocilizumab [138].

Perspectives of clinical trials were presented in a recent review, enumerating the potential therapeutics capable of reducing inflammation, and pointing out the blockade of monocyte costimulators, such as CCR5 (Leronlimab, Emapalumab), which may regulate monocyte and T cell migration to the infected tissue. Additionally, trials evaluating additional antibodies to inhibit IL-1*β* (Anakinra), IFN-*γ*, and myeloid-derived inflammatory cytokines (GM-CSF/anti-GM-CSF) have been described [139, 140].

Using a bioinformatics tool strategy to search for promising compounds targeting immune responses, Richardson et al. in 2020 described that Baricitinib/Ruxolitinib, used for JAK/STAT inhibition and promoting impairment of signaling transduction on cell immune response, may be promising for COVID-19 treatment [141]. Ongoing clinical trials are using JAK inhibitors to evaluate their efficiency in trying to control the cytokine storm, limiting the cytokine expression by immune cells [140, 142].

The complement system plays an important role in the initial cascade of the inflammation process in viral infections, as well as in the production of cytokines/chemokines [143, 144]. It is not different in SARS-CoV-2 infection, in which platelets and complement components may be responsible for the thrombosis observed in some COVID-19 patients [101]. A commercial anti-C5 monoclonal antibody (Eculizumab), presented preliminary results with four patients in the intensive care unit (ICU) with COVID-19, in which all patients recovered after the treatment, diminishing the C-reactive protein and shortening the period of hospitalization [145].

A case report study, treating a child with Crohn's disease and affected by COVID-19, used anti-TNF (Infliximab) with positive results, demonstrated by an improved cytokine profile, with normalization of TNF-*α*, and a decrease of IL-6 and IL-8 concentrations in blood. These preliminary findings support a role for the blockade of TNF-*α* in the treatment of the COVID-19 inflammatory cascade, with possibilities extending to the use of other TNF treatments ((Infliximab, Adalimumab, Golimumab)/Fab′-PEG (Certolizumab) Fusion TNFR2-IgG1-Fc (Etanercept)) [146] (Table 1).

Wang et al. in 2020 performed *in vitro* assays showing that CD147 detected on the surface of lymphocytes and Vero cell lines can be an alternative entry for SARS-CoV-2 in host cells [147]. Koch et al. in 1999 showed that CD147 is a broadly expressed cell surface glycoprotein, whose expression is upregulated upon T cell activation [148]. Grifoni et al. in 2020 showed T cell activation by the SARS-CoV-2 protein in patients that recovered from COVID-19 and in healthy subjects with a history of human common cold coronaviruses [17]. Probably, T cell activation can facilitate the entry of SARS-CoV-2 into T cells through CD147 expression, becoming easily disseminated to other tissues through blood circulation. According to those findings, Wang et al. in 2020 also showed that an anti-CD147 monoclonal antibody could inhibit SARS-CoV-2 replication using *in vitro* cell cultures, being useful for near-future therapies [147]. Currently, a clinical trial is ongoing using a humanized anti-CD147 (Meplazumab) with a potential beneficial effect on COVID-19 treatment [149].

Some review studies have mentioned the use of recombinant human Angiotensin-converting enzyme 2 (rhACE2) to block the entry of SARS-CoV-2 in susceptible cells, contributing to the improvement and control of the immune response during the viral disease. The evaluation of this immunotherapy showed good results using *in vitro* cell culture and in clinical trials [111, 150].

#### 3.1.5. Anticoagulants and Their Effects on Immune Events

Anticoagulant compounds play an important role in preventing the consumption of multiple clotting factors in disseminated intravascular coagulation (DIC) [80], as observed in COVID-19 [67, 151, 152]. Anticoagulant heparin was used in a few patients with severe COVID-19, with important findings on the D-dimer elevation levels and high platelet count, but without presenting any difference with regard to poor outcomes. A large sample size is needed to confirm the effects of heparin in the management of COVID-19 patients [72, 111].

Other compounds in use for hemostatic disorders were observed in COVID-19 patients. Crizanlizumab, a humanized monoclonal antibody to P-selectin recently approved for use in patients with sickle cell anemia, has been pointed as a therapeutic approach. P-selectin is related to the platelet function to maintain hemostatic homeostasis, being an essential cofactor for the extrinsic pathway of blood coagulation. Neri et al. in 2020 described that P-selectin plays a role in leukocyte recruitment into the lungs during SARS by other studies using animal models and cell culture thus suggesting that the monoclonal antibody is a possible therapy to treat COVID-19 [103]. There is an ongoing clinical trial testing this monoclonal antibody on COVID-19 patients (Table 1(b)).

#### 3.1.6. Antibiotics with Immunomodulatory Effects

Antibiotics are normally used as an adjuvant in therapy with antiviral drugs and immunomodulatory components to avoid secondary infections, such as bacterial and fungal infections in hospitalized patients. Despite their antimicrobial function, antibiotics like Azithromycin present immunomodulatory properties, which can reduce inflammatory macrophage polarization and inhibit NF-*κ*B signaling pathways, minimizing the hyperinflammation damage. Since the beginning of COVID-19 treatment, antibiotics have been used with good results in mortality reduction and shortening of intubation time [142, 153].

#### 3.1.7. Immune Cell-Based Therapy

Multipotent mesenchymal stem cells (MSC) derived from placenta, bone marrow, umbilical cord, or adipose tissue have been highlighted in the tissue engineering field to treat several diseases with immunomodulatory properties. Nonclinical studies have shown the efficacy of MSC in the treatment of respiratory diseases, reducing pulmonary edema, decreasing circulating proinflammatory cytokines, and improving mortality rates. On COVID-19 patients, MSC therapy was demonstrated to be effective using allogeneic MSC transplantation without adverse effects in seven patients [111, 154].

As mentioned here, NK cells play an important role in the innate immunity during COVID-19, and their counts dropped in infected patients. Given these findings, NK cell-based immunotherapy is one option to treat COVID-19. It has been under phase I of clinical trials, and companies are aiming at repurposing their anticancer NK-based products, in which they have developed placenta hematopoietic stem cell-derived NK cells, to COVID-19 treatment. Therefore, this appears to be a promising immunotherapy for the near future [111, 150].

Regarding the context of immune cell-based therapy, some authors described the possible use of CAR-T cell therapy to treat COVID-19 patients, to compensate for the loss of T lymphocytes, to help in the elimination of infected cells. Nevertheless, there are some concerns about patient selection, and guidelines should be evaluated in each case to use this therapy. It has demonstrated effectiveness in cancer therapy, and the issues involved with the treatment of COVID-19 are related to other adverse events. Once the CAR-T cell therapy is initiated, the process cannot be aborted [155, 156]. However, no evidence of the success of these products in the management of COVID-19 treatment has been disclosed yet (Table 1).

#### 3.1.8. Biomolecule Effect on Immune Response

In acute lung injury, like SARS, it has been discussed that excessive neutrophil accumulation in the lung tissue that induces severe damage through the release of necrotic cell contents contributes to hyperinflammation and hypoxia. Biomolecules, including compounds from Traditional Chinese Medicine, vitamin C, vitamin D, zinc, melatonin, and nitric oxide are reported to relieve those processes, being promising immunomodulator candidates in immunotherapy against COVID-19 [111, 157].

A study reported that vitamin D deficiency that is not sufficiently supplied is associated with COVID-19 risk [158]. Parikh et al. in 2020 showed that spontaneously breathing patients with COVID-19, who underwent therapy with inhaled nitric oxide (iNO), showed good results and no need for mechanical ventilation [159]. Their findings suggested that iNO therapy may play a role in preventing the progression of hypoxic respiratory failure. Other biomolecules related to good results in respiratory infections are also considered being promising in helping with COVID-19 recovery in combined therapies. Clinical trials are underway aimed at evaluating their effects.

### 3.2. Nonclinical Studies: State of the Art from Laboratory Findings and Other Diseases Related to Host Immune Disorders

#### 3.2.1. Antiviral Compounds with Immunomodulatory Effects

A hepatitis C drug, Daclatasvir, is able to block hepatitis C nonstructural viral protein activities, essential for viral replication in the cell hosts. Sacramento et al. in 2020 demonstrated an inhibitory activity of Daclatasvir for SARS-CoV-2 using an *in vitro* cell culture system, showing similarities in antiviral activity for nsp12 protein, with a reduction of proinflammatory cytokines (IL-6 and TNF-*α*) in early events [160]. In addition, there is no current clinical trial for this drug carried out with COVID-19 patients (Table 2).

#### 3.2.2. Antiviral and Immunomodulatory Effects of Biomolecules: Bovine Lactoferrin

Lactoferrin (Lf) is part of the transferrin family, being an iron-binding protein, which is present in several external secretions such as milk, semen, saliva, mucous secretions, pancreatic fluids, and gastrointestinal secretions [161, 162]. A recently published observational study using bovine lactoferrin in liposomal form showed a decrease in typical symptoms of COVID-19 in 75 patients. In this study, liposomal bovine lactoferrin (32 mg) was administered in the form of syrup together with vitamin C (12 mg) and zinc (10 mg) [163]. Regarding the antiviral activity of lactoferrin, a pioneer study using SARS pseudovirus demonstrated that lactoferrin was able to inhibit the entry of the virus by binding to heparin sulfate proteoglycans (HSPGs), preventing binding of the virus [164]. This same mechanism has demonstrated an inhibition of approximately 80% for Mayaro infection [165]. In that study, the authors demonstrate that the binding of lactoferrin to the Vero cells was highly dependent on HSPG sulfation. This mechanism strongly suggests that inhibition of the Mayaro virus infection occurs by blocking these molecules on the cell surface. Similar results were shown for Zika and Chikungunya virus [166]. More recently, lactoferrin has been shown to be able to inhibit about 85% of SARS-CoV-2 infection in Vero cells [167].

Another recent study, using the influenza virus, showed the action of bovine lactoferrin on the viral particle. The results showed an interaction between lactoferrin and viral hemagglutinin at low pH, which led to the stabilization of this protein, thus preventing the conformational changes required for the virus-cell interaction and consequently the beginning of the viral cycle [168]. In the immunological context of lactoferrin action, it is known that it has an anti-inflammatory action, produced mainly by macrophages and neutrophils, acting in several cascades of cell signaling [169, 170]. Although the bioavailability of iron is fundamental for several metabolic processes, free forms of this ion can be potentially harmful by inducing the formation of reactive oxygen species (ROS). Due to the ability of lactoferrin to bind several ions, including iron, it can act as a chelator for this ion, decreasing its availability and consequently decreasing the inflammatory process by controlling iron homeostasis [169, 171]. In addition, lactoferrin has the ability to control the translation and release of inflammatory molecules, such as IL-1*α*, IL-1*β*, IL-6, IL-8, TNF-*α*, IFN-*α*, IFN-*β*, and other chemokines, with the function of activating the adaptive immune response [169, 172, 173]. Although more studies are needed to reveal the action of lactoferrin in more detail, the therapeutic potential of this protein against COVID-19 is very promising (Table 2).

#### 3.2.3. Inhibition/Blockade of Immune Response Pathways

Based on autoimmune diseases and other infections experienced [174–176], several studies focused on inhibitors or blockers of cellular immune response pathways had been directed to therapeutic targets for COVID-19 treatment, aiming at diminishing the hyperinflammation process led by viral infection. Some authors had mentioned the use of TNF-*α* inhibitors for COVID-19 treatment [175, 176]. McDermott et al. in 2016 used Kepi, an inhibitory subunit of the protein phosphatase 1 complex (PP1), which was managed in mice models infected with SARS-CoV to reduce signaling of inflammatory cytokines such as TNF-*α* [176].

Twelve commercial compounds, including anti-inflammatory drugs, immunomodulators, IFNs, and selected antiviral agents were used by Barnard et al. in 2006 to study their effects on SARS-CoV infection in *in vitro* cell cultures, as well as in Balb/c mice models. The results showed that only hybrid IFN-*α* B/D was effective in inhibiting SARS-CoV replication in Vero cells, by *in vitro* assays, and could reduce SARS-CoV replication in the lungs of infected mice. Nevertheless, the authors raised the concern that infected mice lost significant amounts of weight when treated with IFN. Considering that IFN may contribute to the tissue degradative effects of TNF-*α* already induced by SARS-CoV infection, the problem is to polarize the mouse immune system to a Th2 inflammatory response, with undesired consequences to patient recovery from SARS-CoV infection when treated with an IFN. Thus, more information regarding IFN-*α* B/D treatment for SARS is necessary. Consequently, its use is not yet recommended for COVID-19 treatment [177].

Monocytes (CD14+) require the chemokine receptor CCR2 to exit the bone marrow driven to inflamed tissues, where they accumulate as macrophages. Since this receptor can be blocked, COVID-19 can benefit from reducing the accumulation of macrophages in inflamed lung tissue. Adding to that, the oxidative stress caused by the virus in macrophages could be reduced using an IRAK4 inhibitor, which could influence the molecular mechanisms of TLR7 activation as well as IL-1*β* signaling [140] (Table 2).

#### 3.2.4. Anticoagulants and Their Effects on Immune Events

A study using a clinically approved PAR-1 antagonist—SCH530348—demonstrated that it was able to reduce levels of proinflammatory cytokines, neutrophilic lung inflammation, and alveolar leakage during bacterial pneumonia in a murine model [178]. In another study, it was observed that the activation of PAR-1 contributed to the pathogenesis and worsened survival in mice challenged with the influenza virus (IAV), while treatment using an antagonist of this receptor—SCH79797—was able to protect the mice from IAV infection [179]. These reports, thus, reinforce the theory that inhibition of PAR receptor activation may be an interesting approach for the treatment of microthrombosis and other harmful pathological effects associated with the disseminated intravascular coagulation observed in COVID-19. Nevertheless, the PAR-1 antagonist has any disclosure with COVID-19 yet (Table 2).

#### 3.2.5. Immunotherapeutic Approaches with Similarities to Other Immunopathological Diseases

Chong et al. in 2020 suggest the use of Bruton's tyrosine kinase inhibitor (BTKi), a major kinase in the B-cell receptor signaling pathway, mediating B-cell expansion and apoptosis and used in the management of cancer related to B-cell malignancies, for COVID-19 treatment. The authors assumed that BTKi may present pros and cons for patients with cancer affected by COVID-19 because the product plays an active role in macrophage polarization, downstream of classic M1 and M2 polarizing stimuli, and mitigates the inflammatory response in COVID-19. On the other hand, it offers a risk to secondary infections and impairment of humoral immunity. Given these findings, the authors considered the use of BTKi for the treatment of COVID-19 in patients with B-cell malignancies [180, 181].

Based on previous studies with influenza and lipopolysaccharide antigens, Thomas et al. in 2020 published a hypothesis which revealed that a low molecular weight fraction of commercial human serum albumin (LMWF5A), a novel biological development for osteoarthritis, was able to induce *in vitro* immunomodulatory effects, reducing the cytokine storm and restoring homeostasis to the immune response, attenuating the hyperinflammation, suggesting its positive effects to treat COVID-19 [182] (Table 2).

## 4. Conclusion

Severe acute respiratory syndrome 2 coronavirus (SARS-CoV-2) has been the new challenge for social, economic, and public health systems worldwide. Since March 2020, the World Health Organization (WHO) and other medical institutes have created, invested, and motivated programs and clinical trials to move forward as fast as possible, to provide any results regarding therapeutic possibilities for COVID-19 (https://www.who.int/dg/speeches/detail/who-director-general-s-opening-remarks-at-the-media-briefing-on-COVID-19---11-march-2020). Despite the race for advances in science and new breakthroughs, there is no efficient current therapy for COVID-19. The immunotherapies described here were also administered in combined treatments carried out in human clinical trials. Vaccine development is underway, but tests are needed to guarantee the safety and efficacy and avoid new waves of contamination in the future.

**Figure 4:**
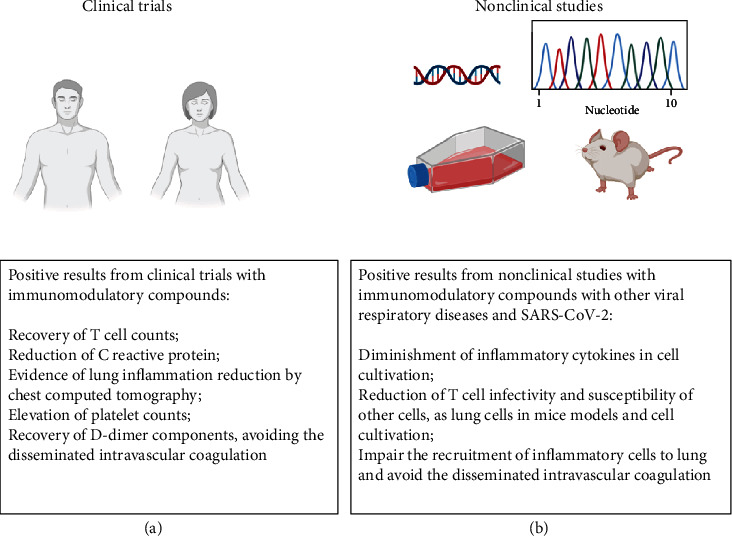
Good results of immunotherapies on clinical trials and nonclinical studies in the immune response against COVID-19. (a) Recent and preliminary positive findings with clinical trials using immunotherapy for COVID-19 treatment are summarized focusing on immune event alterations and recovery, as well as in nonclinical studies (b) for immunotherapeutic interventions using bioinformatics tools, cell cultivation, and animal models; they are also based on past information with other related viral respiratory diseases and autoimmune disorders. Created with BioRender.com.

Here, we were able to summarize important findings in clinical trials and nonclinical studies considering the immunological approaches that could lead to the improvements of COVID-19 therapies, aiming at regulating the cellular immune dysfunction caused by this viral infection with high mortality rates (Figure 4). It is noteworthy to point out that knowledge about the disease is growing daily, with published and unpublished manuscripts (preprint platforms) showing an effort to elucidate mechanisms and understand the pathogenesis as well as propose novel therapeutic targets and strategies. It is also important to emphasize that management of COVID-19 treatment, either immunotherapeutics or not, should be performed within ethical rules and caution, following WHO guidelines and medical practice guidelines, even for *in vitro* and animal *in vivo* purposes. Finally, those scientific findings presented here will be helpful in offering substantial information for physicians, clinicians, and researchers to speed up the process towards the achievement of potential breakthroughs in the near future.

## Figures and Tables

**Table:** (a) Clinical trials with result disclosures

Product	Benefits for COVID-19	ClinicalTrials.gov reference number	Reference
Dexamethasone/methyl prednisolone	Corticosteroids: reduce hyperinflammation and mortality rates	NCT04445506; NCT04323592; NCT04244591; NCT04273321; NCT04374071	Gangopadhyay et al./Chroboczek et al./Ledford/Wang et al. [131–134]
Convalescent plasma	Antibodies from plasma: reduce viral load, days of hospitalization, and the number of deaths. Increase of effector T cell responses helping in viral elimination	NCT04407208; NCT04346446; NCT04441424; NCT04442958	Vabret et al./Shang et al. [30, 108]
Chloroquine and hydroxychloroquine	Antiviral compounds with immunomodulatory effect: without proven effectiveness or potential benefit in COVID-19	NCT04307693; NCT04261517; NCT04434144; NCT04345861; NCT04376814; NCT04343768; NCT04423991; NCT04389320; NCT04441424; NCT04308668; NCT04323592; NCT04306497; NCT04362332; NCT04342650; NCT04323527; NCT04358068; NCT04321278; NCT04333654; NCT04475588; NCT04453501; NCT04308668; NCT04304053	Vabret et al./Zhang et al. [30, 111]
Ivermectin	Antiviral compounds with immunomodulatory effect: reduce mortality rates. Possible effect on raising antibody production and leukocytes	NCT04343092; NCT04434144	Rajter et al. [114]
Oseltamivir	Antiviral compounds with immunomodulatory effect: reduce the hospitalization days for COVID-19 patients	NCT04349241	Coenen et al. [119]
Interferon *β*-1b	Cytokine-directed therapy: combined with other antivirals, reduces the duration of viral shedding, days of hospitalization and mitigation of symptoms	NCT04343768; NCT04276688	Shalhoub [125]
Anti-IL-6 and anti-IL-6R (Tocilizumab/Siltuximab)	Inhibition/blockade of immune response pathways: combined with other antivirals, presented resolution on lung opacities on chest computed tomography and hospital discharge using tocilizumab	NCT04331795; NCT04346355; NCT04322188	Xu et al. [138]
Heparin	Anticoagulants: a preliminary study with D-dimer elevation levels, high platelet count in severe COVID-19	NCT04412304	Zhang et al. [111]
Azithromycin	Antibiotics: used as an adjuvant in therapies with antiviral drugs and immunomodulatory components to avoid secondary infections in hospitalized patients, with good results in mortality reduction and intubation shortening time	NCT04345861; NCT04434144; NCT04343092; NCT04441424; NCT04358068; NCT04321278; NCT04453501	Saghazadeh and Rezaei/Falsenstein et al. [142, 153]
Multipotent mesenchymal stem cells (MSC)	Immune cell-based therapy: results showed a reduction of pulmonary edema and decreased circulating proinflammatory cytokines and mortality rates	ChiCTR2000029990	Zhang et al./ Leng et al. [111, 154]
Vitamin D, zinc, Traditional Chinese Medicine	Biomolecule effect on immune response: contributing to the reduction of hyper inflammation	NCT04407572; NCT04435119; NCT04306497	Zhang et al./Zhang et al./Meltzer et al. [111, 157, 158]

**Table:** (b) Ongoing clinical trials

Product	Benefits for COVID-19	ClinicalTrials.gov reference number	Reference
Intravenous gamma globulin (IVIG)	Antibodies from plasma: may suppress inflammatory cytokines and modulate T cell responses	NCT04411667; NCT04403269; NCT04400058	Zhang et al. [111]
Anti-CCR5 (Leronlimab)	Inhibition/blockade of immune response pathways: may promote monocyte and T cell recruitment in tissues in COVID-19	NCT04347239; NCT04343651	Merad and Martin [140]
Recombinant IL-1 receptor antagonist (Anakinra)	Inhibition/blockade of immune response pathways: may reduce proinflammatory cytokines in COVID-19, as seen in patients with rheumatologic disorders	NCT04339712; NCT04324021; NCT04330638; NCT04443881; NCT04362943; NCT04412291; NCT04364009; NCT04357366; NCT04408326; NCT02735707; NCT04374539; NCT04278404; NCT04462757	Muskardin [139]
Anti-IFN-*γ* (Emapalumab)	Inhibition/blockade of immune response pathways: may reduce pro-inflammatory cytokines in COVID-19	NCT04324021	Merad and Martin [140]
Anti-GM-CSF (Lenzilumab)/GM-CSF (Sargramostim)	Inhibition/blockade of immune response pathways: may reduce proinflammatory cytokines, and promotes macrophage differentiation and survival driving to tissue repair in lungs in COVID-19	NCT04351152; NCT04326920; NCT04400929	Merad and Martin [140]
JAK/STAT inhibition (Baricitinib/Ruxolitinib)	Inhibition/blockade of immune response pathways: may induce impairment of the signaling transduction on cell immune response using bioinformatic tools	NCT04390464; NCT04321993NCT04334044; NCT04351503; NCT04377620; NCT04362137; NCT04338958; NCT04348695; NCT04403243; NCT04359290; NCT04477993	Richardson et al. [141]
Anti-C5 (Eculizumab)	Inhibition/blockade of immune response pathways: diminishing C reactive protein and shortening period of hospitalization	NCT04346797; NCT04351503	Diurno et al. [145]
Anti-TNF (Infliximab)	Inhibition/blockade of immune response pathways: cytokine profile improved with normalization of TNF-*α*, a decrease in IL-6, and IL-8 concentrations	NCT04425538; NCT04344249	Dolinger et al. [146]
Anti-CD147 (humanized Meplazumab)	Inhibition/blockade of immune response pathways: inhibition of SARS-CoV-2 entry and replication using *in vitro* cell cultures, may reduce T cell activation and T cell infectivity. Reduce viral replication in humans with COVID-19	NCT04275245	Wang et al./Ulrich and Pillat [147, 149]
rhACE2	Inhibition/blockade of immune response pathways: inhibition of SARS-CoV-2 entry and replication as well as may control immune cell response	NCT04335136	Zhang et al./Tu et al. [111, 150]
Natural killer cells (NK)	Immune cell-based therapy: may help in elimination of infected cells	NCT04365101; NCT04280224NCT04324996; NCT04375176	Zhang et al./Tu et al. [111, 150]
Biomolecules: vitamin C, melatonin, nitric oxide (iNO)	Immunomodulatory effects of biomolecules: may contribute to reducing hyperinflammation and prevention of hypoxic respiratory failure	NCT04323514; NCT04357782; NCT04370288; NCT04328961; NCT04354428; NCT04264533; NCT04409522; NCT04353128; NCT04446104; NCT04326725; NCT04360980; NCT04368897; NCT04388683; NCT04338828; NCT04305457; NCT04337918; NCT04306393; NCT04312243; NCT04445246; NCT04334512; NCT03680274; NCT04335084; NCT04468139; NCT04421508; NCT04397692	Zhang et al./Tu et al./Zhang et al./Parikh et al. [111, 150, 157, 159]
Atazanavir	Antiviral compounds with immunomodulatory effect: reduce viral load and proinflammatory cytokines using *in vitro* cell cultivation with SARS-CoV-2	NCT04452565	Merad and Martin [140]
Humanized monoclonal antibody to P-selectin (Crizanlizumab)	Anticoagulants: anti-P-selectin may help to maintain the hemostatic homeostasis, being an essential cofactor for the extrinsic pathway of blood coagulation	NCT04435184	Neri et al. [103]
CAR-T cell therapy	Immunotherapeutic approaches with similarities to other immunopathological diseases: may help the elimination of infected cells	NCT04474067	Bachanova et al./Ljungman et al. [155, 156]

**Table 2:** Nonclinical studies focused on immunotherapy with potential benefits to COVID-19 treatment.

Product	Potential benefits for COVID-19	Reference
Daclatasvir	Antiviral compounds with immunomodulatory effect: reduce viral load and proinflammatory cytokines using *in vitro* cell cultivation with SARS-CoV-2	Sacramento et al. [160]
Bovine lactoferrin	Antiviral compounds with immunomodulatory effect: reduce viral load and possible reduction of proinflammatory cytokines and induction of T cell activation	Carvalho et al. [167]
Kepi—inhibitory subunit of the protein phosphatase 1 complex (PP1)	Inhibition/blockade of immune response pathways: reduce signaling of inflammatory cytokines, as TNF-*α* in mice models infected with SARS-CoV	McDermott et al. [176]
Hybrid IFN-*α* B/D	Inhibition/blockade of immune response pathways: it was effective to inhibit SARS-CoV replication in Vero cells by *in vitro* assays and could reduce SARS-CoV replication in the lungs of infected mice	Barnard et al. [177]
CCR2 antagonist/anti-CCR2	Inhibition/blockade of immune response pathways: may promote monocyte egress from the bone marrow and monocyte recruitment in tissues	Merad and Martin [140]
IRAK4 inhibitor/PF-06650833; CA-4948	Inhibition/blockade of immune response pathways: may reduce inflammation caused by macrophages, mediates TLR and IL-1*β* signaling	Merad and Martin [140]
PAR-1 antagonist (SCH530348), PAR-1 antagonist receptor (SCH79797)	Anticoagulants: may reduce levels of proinflammatory cytokines, neutrophilic lung inflammation, and other harmful pathological effects that are associated with the disseminated intravascular coagulation in COVID-19	Khoufache et al./Bacil et al. [179, 183]
Inhibitor of Bruton's tyrosine kinase (BTKi)	Immunotherapeutic approaches with similarities to other immunopathological diseases: may reduce B cell responses and macrophage polarization, as observed in patients with B cell malignancies	Chong et al. [180]

## Data Availability

No data were used to support this study. The figures and table data used to support the findings of this study are included within the article.
